# Association between parity and obesity patterns in a middle-aged and older Chinese population: a cross-sectional analysis in the Tongji-Dongfeng cohort study

**DOI:** 10.1186/s12986-016-0133-7

**Published:** 2016-10-26

**Authors:** Wending Li, Yi Wang, Lijun Shen, Lulu Song, Hui Li, Bingqing Liu, Jing Yuan, Youjie Wang

**Affiliations:** 1 School of Public Health, Tongji Medical College, Huazhong University of Science & Technology, Hangkong Road 13, Wuhan, 430030 China; 2 Department of Maternal and Child Health, School of Public Health, Tongji Medical College, Huazhong University of Science & Technology, Wuhan, China; 3 Ministry of Education Key Lab for Environment and Health, School of Public Health, Tongji Medical College, Huazhong University of Science and Technology, Wuhan, China

**Keywords:** General obesity, Abdominal obesity, Parity, Childbirth, Chinese

## Abstract

**Background:**

Higher parity has been implicated as a risk factor for obesity of women. The objective of the study was to examine whether parity was associated with general obesity or abdominal obesity, or both, among middle-aged and older Chinese women.

**Methods:**

A total of 12,829 Chinese women (mean age: 64.8 years) with at least one live birth were selected from the Dongfeng–Tongji Cohort Study (phase II). We used body mass index to assess general obesity, and waist-to-hip ratio (WHR), waist-to-height ratio (WHtR) and waist circumference (WC) to assess abdominal obesity. We used multivariate linear and logistic regression models to investigate the association between parity and obesity.

**Results:**

The values of all four obesity measures increased with the greater number of live births (*P* for trend <0.001). After adjustment for potential confounders, women with four or more children had 1.72 times (95 % confidence interval [CI], 1.41–2.10) higher risk of general obesity, and 1.93 (95 % CI, 1.57–2.37), 2.09 (95 % CI, 1.65–3.64) and 1.58 (95 % CI, 1.28–1.94) times risk of abdominal obesity assessed by WHR, WHtR and WC, respectively. Furthermore, we observed an ascending gradient between parity and the three abdominal obesity measures.

**Conclusions:**

Parity was positively associated with risk of obesity, especially abdominal obesity, in the long term among Chinese women.

**Electronic supplementary material:**

The online version of this article (doi:10.1186/s12986-016-0133-7) contains supplementary material, which is available to authorized users.

## Background

Obesity has been increasing globally, and the World Health Organization (WHO) reported that in 2014, 39 % of adults aged 18 years and older were overweight. As a fast-developing country, China is facing an upsurge in obesity [1]. Between 2004 and 2009, a nationwide study found that more than two in five middle-aged Chinese were overweight or obese (body mass index [BMI] ≥24.0 kg/m^2^, Chinese criteria), suggesting an urgent need to better understand the causes of obesity [2].

Pregnancy involves physiological and psychological changes, and may induce insulin resistance in peripheral tissues [3], weight gain or obesity, and postpartum weight retention [4]. Although multiple studies have reported a positive association between parity and obesity, there is controversy with the type of obesity (general or abdominal), the level of parity (primiparous or multiparous), and the strength, trigger time and length of time of the association. A study of Chilean women concluded that parity moderately influenced BMI, but was unrelated to abdominal obesity [5]. Other studies have indicated that abdominal obesity, but not BMI, is significantly related to increased parities [6, 7].

In some prospective studies, increased BMI was only observed after the first childbirth and not after later childbirths [8, 9], whereas other studies have suggested a positive gradient with consecutive pregnancies [5, 7]. A 7-year follow-up study found that childbearing might not increase the incidence of obesity among parous young women in the USA [10]. Additionally, a meta-analysis investigation reported a U-shaped secular trend of postpartum weight retention for women who gained excess weight during pregnancy, indicating that in addition to short-term obesity, women were also at greater risk of obesity over the long term [11]. However, contrary to this finding, a nationwide cohort study in the USA observed significant parity-related weight gain in a 10-year follow-up, but not after 25 years [8], suggesting that long-term correlation requires further confirmation.

Studies have revealed that most Asians (Chinese, Indonesians and Thais) have a higher percentage of body fat for a given BMI than Europeans [12]. In addition, Asians are genetically more susceptible to morbidities that include the accumulation of visceral fat (e.g. metabolic syndrome, coronary heart disease, and diabetes) [13]. Therefore, evaluation of abdominal obesity, rather than other forms of obesity, would be more meaningful among an Asian population [14]. Current evidence suggests that the parity-obesity association varies among different cultures [15], ethnic groups [6, 8, 9, 11] and levels of country development [16]. There has been no previous research on the relationship between obesity and number of children among Chinese population. We aimed to examine whether parity was associated with general obesity or abdominal obesity, or both, among middle-aged and older Chinese women.

## Methods

### Study population

This analysis used data from the Dongfeng–Tongji cohort study (phase II), which was launched in 2013 among retirees of the Dongfeng Motor Corporation (DMC) in Shiyan, Hubei Province, China. Details of the Dongfeng–Tongji cohort design, fundamentals, and methods have been previously described [17]. A total of 38,295 retired DMC employees agreed to participate in the Dongfeng–Tongji cohort study (phase II). Each participant was required to complete a standard questionnaire via a face-to-face interview, undergo a medical examination, and provide a blood sample. Exclusion criteria included all men, women with missing data for parity or obesity measurements (weight, height, waist circumference and hip circumference), and nulliparous women. The final study population included 12,829 participants (mean age: 64.8 ± 7.6 years). Written informed consent was obtained from all participants, and the Medical Ethics Committee of the School of Public Health, Tongji Medical College and Dongfeng General Hospital approved the study.

### Parity

We defined parity as the self-reported total number of live births, which we classified into four categories: one, two, three, and four or more live births.

### Anthropometric measurements

All anthropometric measurements, including weight, height, waist and hip circumference, were carried out with standard apparatus by trained medical staff at hospitals affiliated to DMC. BMI was calculated by dividing weight (kg) by height squared (m^2^). Waist-to-hip ratio (WHR) and waist-to-height ratio (WHtR) were calculated by dividing waist circumference (WC), respectively, by hip circumference and height, and measured both in the same units. In the current study, general obesity was defined as BMI ≥24.0 kg/m^2^ (including 24.0–27.9 kg/m^2^ for overweight and ≥28.0 kg/m^2^ for obesity) using the Chinese cut-off as recommended by the Working Group on Obesity in China [18]. Abdominal obesity was defined as WC ≥ 80.0 cm as recommended by the Working Group on Obesity in China, or WHR ≥ 0.85 as recommended by WHO [19] or WHtR ≥ 0.5 as based on previous studies [20].

### Assessment of covariates

Sociodemographic characteristics including sex, age, education (primary school or below, junior high school, senior high school, college or above), and marital status (married, unmarried, widowed or divorced) were collected from the questionnaire replies. We also obtained lifestyle characteristics including physical activity, smoking and alcohol drinking status from the questionnaires. We obtained reproductive data including menopause status, abortion, the use of contraceptives, and the use of hormone replacement therapy, which were self-reported from the questionnaires. Peripheral venous blood samples were collected after overnight fasting, and plasma glucose levels were measured with Aeroset automatic analyzer (by glucose oxidase method; Abbott Laboratories. Abbott Park, Illinois, USA). We defined diabetes mellitus as fasting plasma glucose ≥7.0 mmol/L, self-reported physician diagnosis of diabetes mellitus, or current use of antidiabetic medications. Similarly, hypertension was defined as a self-reported previous diagnosis of hypertension, taking antihypertensive treatment, or systolic blood pressure >140 mmHg or diastolic blood pressure >90 mmHg.

### Statistical analysis

We summarized numerical data as means ± standard deviation (SD) and presented categorical variables as percentages. We used analysis of variance (ANOVA) or *χ* ^*2*^ test to test the difference among parity groups. We used four hierarchical models to estimate the effect and the risk of increased parity on obesity in both linear and logistic regression. Model 1 examined the relationship between parity and obesity without adjustment for any covariates. Model 2 included age plus parity. Model 3 included the variables in Model 2 plus diabetes and hypertension. Model 4 included the variables in Model 3 plus education level, marital status, physical activity, smoking status (current or passive smoker), current alcohol drinker and current tea drinker, use of contraceptives, hormone replacement therapy, menopause status and abortion. In general linear regression, we calculated the variance inflation factor (VIF) to detect possible multi-collinearity during modeling. We carried out statistical analysis of the data using SPSS statistical software (version 18.0, IBM, Inc.).

## Results

Table 1 presents the descriptive characteristics of the study population. Women with higher parity were more likely to be older, less educated, doing less physical exercise, married or widowed, and current or previous smokers. We also found prevalence of diabetes mellitus, hypertension or menopause to increase with parity. Multiparous women tended to show a lower prevalence of abortion, passive smoking, having a habit of drinking alcohol or tea, or having used contraceptives or hormone replacement therapy.

**Table 1:** The descriptive characteristics of 12,829 retired Chinese women of The DFTJ Cohort, by number of parity

Variable	Parity					
	1	2	3	≥4	*χ* ^2^/F	*P* for trend
	(*n* = 4362)	(*n* = 4410)	(*n* = 2543)	(*n* = 1514)		
Age (years) (mean ± SD)	58.61 ± 4.69	64.84 ± 5.46	69.88 ± 6.01	74.36 ± 5.97	4252.18^**^	<0.001
Education level					2561.64^**^	<0.001
Primary school or illiteracy (%)	415 (9.6)	1184 (27.0)	1116 (44.2)	1022 (68.1)		
Middle school (%)	1742 (40.1)	1858 (42.3)	973 (38.5)	378 (25.2)		
High school (%)	1767 (40.7)	1049 (23.9)	362 (14.3)	77 (5.1)		
College or higher (%)	416 (9.6)	298 (6.8)	73 (2.9)	23 (1.5)		
Marital status					828.61^**^	<0.001
Single (%)	6 (0.1)	3 (0.1)	3 (0.1)	1 (0.1)		
Married or Remarried (%)	3892 (89.7)	3692 (84.0)	1988 (78.6)	990 (65.6)		
Divorced (%)	169 (3.9)	108 (2.5)	22 (0.9)	4 (0.3)		
Widowed (%)	274 (6.3)	593 (13.5)	515 (20.4)	515 (34.1)		
Physical activity (%)	3918 (89.8)	3925 (89.0)	2221 (87.3)	1226 (81.0)	89.25^**^	<0.001
Current/Former smoker (%)	61 (1.4)	105 (2.4)	115 (4.5)	121 (8.0)	184.87^**^	<0.001
Passive smoking (%)	1500 (35.0)	1193 (27.6)	587 (23.6)	333 (22.4)	146.58^**^	<0.001
Current/Former alcohol drinker (%)	548 (12.6)	479 (10.9)	251 (9.9)	124 (8.2)	26.87^**^	<0.001
Current tea drinker (%)	1841 (42.2)	1603 (36.4)	824 (32.4)	403 (26.6)	144.00^**^	<0.001
Diabetes Mellitus (%)	406 (9.3)	662 (15.1)	523 (20.7)	344 (22.8)	222.34^**^	<0.001
Hypertension (%)	1337 (30.7)	1966 (44.6)	1368 (53.9)	888 (58.7)	553.88^**^	<0.001
Ever used Contraceptives (%)	845 (19.5)	885 (20.2)	434 (17.2)	153 (10.2)	83.26^**^	<0.001
Hormone replacement therapy (%)	160 (3.7)	113 (2.6)	52 (2.1)	13 (0.9)	40.64^**^	<0.001
Menopause (%)	4090 (93.8)	4333 (98.3)	2523 (99.3)	1495 (98.7)	238.61^**^	<0.001
Abortion frequency					519.00^**^	<0.001
0 time (%)	1033 (24.3)	1497 (35.1)	1033 (42.1)	772 (53.1)		
1 time (%)	1434 (33.7)	1328 (31.1)	764 (31.1)	364 (25.0)		
2 or more times (%)	1786 (42.0)	1441 (33.8)	658 (26.8)	318 (21.9)		

Numerical data are presented as mean ± SD and tested with F test; Categorical data are presented with percentage in parentheses after the numbers and tested with *χ* ^2^ test

**P < 0.001

**Fig. 1:**
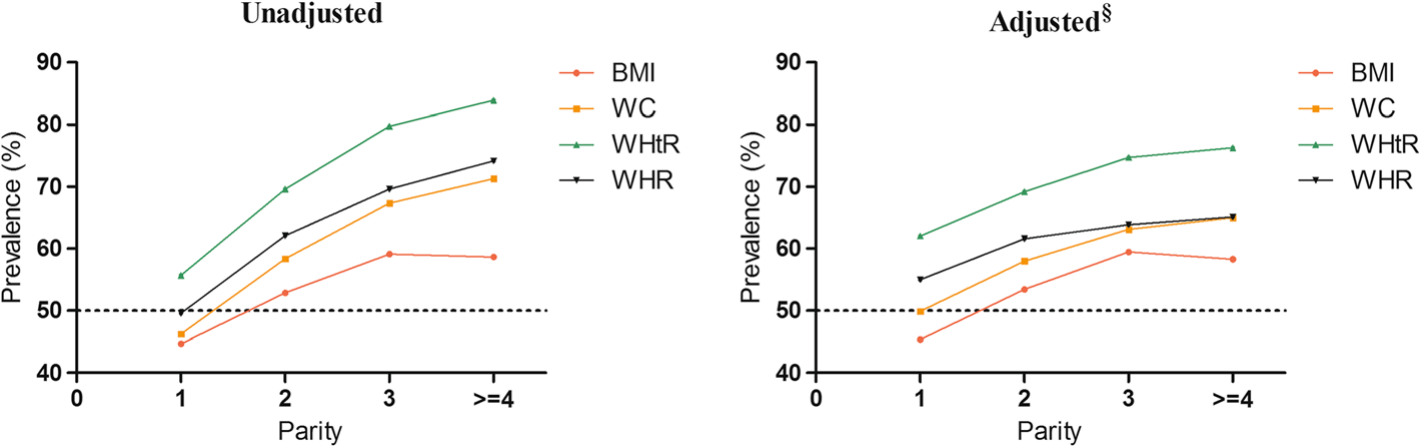
Parity-specific prevalence of obesity by different anthropometric measures. Abbreviations: BMI, body mass index; WC, waist circumference; WHtR, waist-to-height ratio; WHR, waist-to-hip ratio. §: adjusted for age, DM and hypertension, education level, marital status, physical activities, smoking status (current/passive smoker), tea/alcohol drinking status, ever used contraceptives, HRT, menopause status, abortion frequency and gynecologic diseases

The age-adjusted mean values of the four obesity measurements according to parity are shown in Table 2. The mean values of BMI, WC, WHtR and WHR showed an increasing trend with higher parities (*P* for trend <0.001). The obesity measurements all showed that the prevalence of obesity increased with parity before or after adjusting for all potential variables (Fig. 1). BMI-delimited obesity had generally lower prevalence rates with or without adjustment, while WHtR predicted the highest obesity prevalence rates.

**Table 2:** Age-adjusted means (95 % CI) of four measurements of obesity according to number of parity

Measurement	Parity				*P* for trend
	1 (*n* = 4362)	2 (*n* = 4410)	3 (*n* = 2543)	≥4 (*n* = 1514)	
BMI (kg/m^2^)	23.79 (23.66, 23.92)	24.47 (24.36, 24.57)	25.18 (25.03, 25.34)	25.18 (24.96, 25.40)	<0.001
WC (cm)	80.79 (80.45, 81.12)	82.80 (82.52, 83.08)	84.30 (83.90, 84.70)	85.03 (84.45, 85.60)	<0.001
WHtR	0.520 (0.518, 0.522)	0.534 (0.532, 0.535)	0.545 (0.542, 0.547)	0.551 (0.547, 0.555)	<0.001
WHR	0.857 (0.855, 0.859)	0.868 (0.866, 0.870)	0.875 (0.872, 0.878)	0.882 (0.878, 0.886)	<0.001

Abbreviations: *BMI* body mass index, *WC* waist circumference, *WHtR* waist-to-height ratio, *WHR* waist-to-hip ratio

Table 3 presents the results of linear regression from the four models, in which parity was considered as a continuous variable. The VIF was introduced to detect possible multi-collinearity during modeling. The two variables with the highest VIF values in Model 4 were parity (VIF = 2.18) and age (VIF = 2.13). However, neither surpassed the threshold of 10, suggesting a less likely multi-collinearity in our modeling [21]. Regression coefficients for parity calculated as explanatory variables were added into the four models successively. The results showed that after adjustment for the potential confounders, all four measurements of obesity were significantly associated with parity (all *P* <0.05). The fully adjusted β-coefficient of parity for BMI, WC, WHtR and WHR were 0.34, 0.97, 0.0063 and 0.0050, respectively.

**Table 3:** β-coefficients (95 % CI) for parity and different measurements of obesity

	Model 1	Model 2	Model 3	Model 4
BMI	0.38 (0.35, 0.41)	0.48 (0.44, 0.52)	0.45 (0.41, 0.49)	0.34 (0.29, 0.39)
WC	1.77 (1.69, 1.85)	1.40 (1.29, 1.51)	1.32 (1.21, 1.43)	0.97 (0.85, 1.09)
WHtR^a^	1.54 (1.49, 1.59)	1.01 (0.93, 1.09)	0.96 (0.89, 1.03)	0.63 (0.55, 0.71)
WHR^a^	1.18 (1.13, 1.23)	0.76 (0.69, 0.83)	0.72 (0.65, 0.79)	0.50 (0.42, 0.58)
VIF for parity	1.00	1.91	1.92	2.18

Abbreviations: *BMI* body mass index, *WC* waist circumference, *WHtR* waist-to-height ratio, *WHR* waist-to-hip ratio, *VIF* variance inflation factor

^a^β-coefficient amplified by 100

Model 1: unadjusted; Model 2: adjusted for age; Model 3: adjusted for covariate in Model 2 plus DM and hypertension; Model 4: adjusted for covariates in Model 3 plus education level, marital status, physical activity, smoking status (current smoker, passive smoker), current alcohol drinker, current tea drinker, ever used contraceptives, hormone replacement therapy, menopause status and abortion frequency

The crude and adjusted odds ratios (ORs) with 95 % confidence intervals (CI) for different measurements of obesity according to parity are shown in Table 4. In the crude model, parity was significantly associated with risk of all obesity measurements, with abdominal obesity measurements (WC, WHtR and WHR) showing greater OR than general obesity measurements (BMI). In Model 2, OR values of all three abdominal obesity measurements decreased, whereas that of general obesity measurements increased among all three parity groups. In Model 3 and Model 4, all ORs attenuated but remained statistically significant. For women who had four or more children, the obesity rate was 1.72 times higher by BMI, 1.93 times higher by WC, 2.09 times higher by WHtR and 1.58 times higher by WHR than those of monoparous women. From Model 1 to Model 4, we observed a consistent gradient in WC, WHtR and WHR through modeling, but the gradient of BMI receded in the highest parity group, although the general trend was significant.

**Table 4:** ORs (95 % CI) for parity and different measurements of obesity

Measurement	Parity	Model 1	Model 2	Model 3	Model 4
BM ≥ 24.0 kg/m^2^	1	1.00	1.00	1.00	1.00
	2	1.41 (1.28, 1.55)	1.52 (1.36, 1.69)	1.47 (1.32, 1.64)	1.39 (1.24, 1.56)
	3	1.83 (1.63, 2.05)	2.09 (1.82, 2.42)	2.00 (1.73, 2.32)	1.79 (1.54, 2.08)
	≥4	1.77 (1.54, 2.03)	2.14 (1.78, 2.56)	2.09 (1.74, 2.52)	1.72 (1.41, 2.10)
*P* for trend		<0.001	<0.001	<0.001	<0.001
WC ≥ 80 cm	1	1.00	1.00	1.00	1.00
	2	1.69 (1.53, 1.86)	1.55 (1.39, 1.73)	1.51 (1.35, 1.69)	1.41 ^(^1.26, 1.58)
	3	2.44 (2.17, 2.74)	2.11 (1.83, 2.44)	2.03 (1.75, 2.35)	1.76 (1.51, 2.05)
	≥4	3.04 (2.62, 3.53)	2.49 (2.06, 3.01)	2.45 (2.02, 2.97)	1.93 (1.57, 2.37)
*P* for trend		<0.001	<0.001	<0.001	<0.001
WHtR ≥ 0.5	1	1.00	1.00	1.00	1.00
	2	1.89 (1.71, 2.09)	1.60 (1.43, 1.80)	1.57 (1.40, 1.76)	1.41 (1.25, 1.58)
	3	3.19 (2.80, 3.63)	2.39 (2.04, 2.80)	2.31 (1.97, 2.72)	1.88 (1.59, 2.23)
	≥4	4.36 (3.66, 5.21)	2.92 (2.35, 3.64)	2.90 (2.32, 3.62)	2.09 (1.65, 3.64)
*P* for trend		<0.001	<0.001	<0.001	<0.001
WHR ≥ 0.85	1	1.00	1.00	1.00	1.00
	2	1.70 (1.54, 1.88)	1.47 (1.32, 1.64)	1.43 (1.28, 1.60)	1.34 (1.19, 1.50)
	3	2.30 (2.04, 2.59)	1.78 (1.53, 2.06)	1.70 (1.46, 1.97)	1.48 (1.27, 1.73)
	≥4	2.92 (2.51, 3.40)	2.04 (1.68, 2.48)	2.00 (1.64, 2.43)	1.58 (1.28, 1.94)
*P* for trend		<0.001	<0.001	<0.001	<0.001

Abbreviations: *BMI* body mass index, *WC* waist circumference, *WHtR* waist-to-height ratio, *WHR* waist-to-hip ratio

Model 1: unadjusted; Model 2: adjusted for age; Model 3: adjusted for covariates in Model 2 plus DM and hypertension; Model 4: adjusted for covariates in Model 3 plus education level, marital status, physical activity, smoking status (current smoker, passive smoker), current alcohol drinker, current tea drinker, ever used contraceptives, hormone replacement therapy, menopause status and abortion frequency

## Discussion

We found a positive correlation between higher parity and the risk of both general and abdominal obesity in middle-aged and older Chinese women. Furthermore, we observed an ascending gradient between parity and the three abdominal obesity measures.

Most existing studies have reported a positive association between parity and weight gain or BMI [5, 7–9, 16, 22, 23]. However, a few studies have incorporated abdominal measurements and their results have been inconsistent. Mansour et al. [7] reported that higher parity was significantly associated with BMI and all three abdominal obesity measurements among a middle-aged Iraqi population, which was consistent with our study. Another study from Finland concurred with most of our results [23]. They reported a general positive association between parity and BMI and WC, and found that abdominal obesity was more prevalent among multiparous women than with other groups. A prospective study also indicated that childbearing might increase visceral adipose tissue independent of overall increase in body fat [24]. Two studies have partially investigated the relationship between parity and obesity among Chinese middle and older-aged women. Wen et al. [25] reported that weight gain was associated with increasing parity in Shanghai, China, and the study in Guangzhou, China, reported a positive correlation between number of parity and obesity as measured by BMI and WHR [26]. These two studies supported our finding that parity was associated with both general and abdominal obesity among a Chinese population. In addition, abdominal obesity has already been regarded as an important risk factor for metabolic syndrome. Our present data also demonstrated that higher parity was associated with increased risk of obesity related diseases, such as diabetes (see Additional file 1: Table S1). This analysis from the same data supported our findings of association between parity and obesity.

The mechanisms underlying the association between parity and obesity are complicated and remain unknown [11]. Gestational weight gain has been found to be associated with higher postpartum weight retention [27], especially in the long term [28], suggesting that a maternal transition during pregnancy may partly explain postpartum obesity. During pregnancy, the release of placental corticotropin-releasing hormone might quantitatively drive the maternal hypothalamic-pituitary-adrenal axis and cortisol concentrations [29]. Both have been found to play a role in the pathophysiological mechanism of abdominal obesity [30, 31], which may be partly mediated by insulin resistance [32], an important pathway also switched on by pregnancy [3]. Moreover, non-biological disturbance during pregnancy including socioeconomic and psychosocial stress, unhealthy lifestyles and traits of depression and anxiety may also contribute to hypothalamic-pituitary-adrenal hyperactivity [30, 32, 33]. Peripheral insulin resistance triggered by pregnancy resulting in surplus calorie storage might also play an independent role. When the ability of adipose tissue to store the excess energy is limited due to insulin resistance, the triacylglycerol surplus is deposited at undesirable sites such as visceral adipose tissue [34]. This is manifested as an increase in waist girth. These two possible pathways support the conjecture that pregnancy is more likely to induce abdominal obesity.

Nevertheless, lifestyle alterations involving energy intake and expenditure during pregnancy can last a long time post-pregnancy [28], and consecutive repetition of such an alteration, through further pregnancies, may switch the fertile mother’s lifestyle forever. However, Lawlor et al. [35] reported that parity was positively associated with BMI in both sexes, but only influenced WHR among women. This finding suggests that higher parity might induce general obesity in a non-biological manner. This may partly explain why abdominal obesity measurements remained strongly associated with childbirth after adjusting for multiple lifestyle factors as observed in our study.

We excluded nulliparity women from the study for several reasons. First, findings in other populations have generally indicated that parous women compared with nulliparous women have different physiological and pathological characteristics [36]. Childlessness among Chinese women is mainly caused by polycystic ovary disease [37], which may lead to a decrease in ovulatory cycles, alter female hormone levels, and cause an increase in BMI and obesity [38]. Second, pre-existing obesity could induce infertility through an already elucidated mechanism [27]. Therefore, the incorporation of nulliparity into the analysis without prior examination can result in a paradox. For example, Luoto et al. [23] found that women with one or two childbirths had a lower risk of BMI-delimited obesity, while women with three or more childbirths had a higher risk of WC-delimited obesity when compared with a nulliparity group [23].

Our study has several strengths over existing studies of parity and obesity. First, to our knowledge, this is the first comprehensive analysis of parity and obesity among a large population of Chinese women. Second, all three abdominal obesity measurements, which may have different strengths in risk prediction of different comorbidities [39–41], were included in our study. In addition, trained staff using standard procedures carried out anthropometric measurements. Therefore, ascertainment bias was unlikely in our study. Finally, our study had a large sample population and abundant data on demographics, lifestyle, and reproduction-related factors, which not only enhanced the precision, but also allowed statistical adjustment for multiple variables.

There are also some limitations in our study. First, our study design was cross-sectional, which made it difficult to determine the temporal association between parity and obesity. Secondly, although we have made the adjustment for age, the association between parity and obesity might be mediated by age as older women were likely to have more childbirths. But the results of age-matched analysis (see Additional file 1: Table S2) still showed the same relationship, suggesting age was not likely to be a potential confounder. Finally, the participants were a middle-aged and older population, which may have reduced the generalization of this study to other age or ethnic groups. Although we adjusted for multiple covariates and cautiously made conclusions on the long-term influence of childbearing, this made it difficult to control for other possible covariates. Therefore, these results require further corroboration from future prospective studies.

## Conclusions

In conclusion, we found that parity was positively associated with obesity among Chinese middle-aged and older women, and the association was more likely with abdominal rather than general obesity. In China, although younger women tend to have less children, a large proportion of Chinese women, especially those living in the rural or poor area, have two or more children [42]. The findings of this study might help the health professionals to identify women at higher risk of obesity or obesity related diseases for early prevention.

## Additional file

Additional file 1: Table S1. Fully adjusted OR (95 % CI) of metabolism-related diseases, by number of parity. Metabolism-related diseases included diabetes and hypertension. **Table S2.** Means (95 % CI) of BMI/WC/WHR/WHtR difference between multiparous individuals and monoparous individuals, pair-matched by age. Mean differences were calculated and tested at zero using paired *t*-test. For pair matching, age difference was set at less than or equal to 1. (DOCX 29 kb)

## Abbreviations

ANOVA: Analysis of variance
BMI: Body mass index
DMC: Dongfeng motor corporation
SD: Standard deviation
VIF: Variance inflation factor
WC: Waist circumference
WHO: World Health Organization
WHR: Waist-to-hip ratio
WHtR: Waist-to-height ratio
