# Supplementary material for: Association between parity and obesity patterns in a middle-aged and older Chinese population: a cross-sectional analysis in the Tongji-Dongfeng cohort study
Source: Nutr Metab (Lond). 2016 Oct 26;13:72. doi: 10.1186/s12986-016-0133-7 (PMC5081958; doi:10.1186/s12986-016-0133-7)
Supplement: Additional file 1: Table S1. — Fully adjusted OR (95 % CI) of metabolism-related diseases, by number of parity. Metabolism-related diseases included diabetes and hypertension. Table S2. Means (95 % CI) of BMI/WC/WHR/WHtR difference between multiparous individuals and monoparous individuals, pair-matched by age. Mean differences were calculated and tested at zero using paired t-test. For pair matching, age difference was set at less than or equal to 1. (DOCX 29 kb) [file 12986_2016_133_MOESM1_ESM.docx]

Association between Parity and Obesity Patterns in a Middle-aged and Older Chinese Population： a cross-sectional analysis in the Tongji-Dongfeng Cohort study

Wending Li^1^, Yi Wang^1^, Lijun Shen^1,2^, Lulu Song^,2^, Hui Li^2^, Bingqing Liu^2^, Jing Yuan ^3^, Youjie Wang^2,3^

**Additional File 1**

**Author’s E-mail Address:**

Wending Li: [Wending_lee@foxmail.com](mailto:Wending_lee@foxmail.com);

Yi Wang: [wangyi_husttj@163.com](mailto:wangyi_husttj@163.com);

Lijun Shen: [shenyi611613@163.com](mailto:shenyi611613@163.com);

Lulu Song: [sll1234@126.com](mailto:sll1234@126.com);

Hui Li: [1102162682@qq.com](mailto:1102162682@qq.com);

Bingqing Liu: bluebelllbq@163.com;

Jing Yuan: [liangyuan217@163.com](mailto:liangyuan217@163.com);

Youjie Wang: [wangyoujie@mails.tjmu.edu.cn](mailto:wangyoujie@mails.tjmu.edu.cn)

**Author for correspondence:**

Youjie Wang, MD, PhD

School of Public Health

Tongji Medical College

Huazhong University of Science and Technology

Hangkong Road 13, Wuhan, 430030, China

Tel: +86 27 83691198; fax: +86 27 83692701

**Supplementary Table S1:** Fully adjusted OR (95%CI) of metabolism-related diseases, by number of parity

| Disease | Parity | | | |
| --- | --- | --- | --- | --- |
|  | 1 | 2 | 3 | ≥4 |
| Diabetes | 1.00 | 1.41(1.21, 1.65) | 1.83(1.51, 2.23) | 2.02(1.58, 2.57) |
| P: | - | <0.001 | <0.001 | <0.001 |
| Hypertension | 1.00 | 1.12(1.00, 1.25) | 1.15(0.99, 1.33) | 1.07(0.89, 1.28) |
| P: | - | 0.048 | 0.062 | 0.506 |

Fully adjusted for age, education level, marital status, physical activity, smoking status (current smoker, passive smoker), current alcohol drinker, current tea drinker, ever used contraceptives, hormone replacement therapy, menopause status and abortion frequency.

**Supplementary Table S2:** Means (95% CI) of BMI/WC/WHR/WHtR difference between multiparous individuals and monoparous individuals, pair-matched by age#

|  | Means of difference, pair-matched | | |
| --- | --- | --- | --- |
|  | P2 – P1  N=3344 | P3 – P1  N=1878 | P4 – P1  N= 977 |
| ΔBMI | 0.4764 (0.3130, 0.6397) | 0.8276 (0.5971, 1.0580) | 0.6257 (0.2827, 0.9686) |
| t and P value | t=5.72 P<0.0001 | t=7.04 P<0.0001 | t=3.58 P=0.0004 |
| ΔWC | 0.8698 (0.4304, 1.3092) | 1.7775 (1.1912, 2.3638) | 2.0628 (1.2325, 2.8930) |
| t and P value | t=3.88 P=0.0001 | t=5.95 P<0.0001 | t=4.88 P<0.0001 |
| ΔWHR | 0.00194 (-0.00087, 0.00475) | 0.00697 (0.00330, 0.0106) | 0.0159 (0.0108, 0.0211) |
| t and P value | t=1.35 P=0.1762 | t=3.73 P=0.0002 | t=6.10 P<0.0001 |
| ΔWHtR | 0.00468 (0.00190, 0.00747) | 0.0111 (0.00721, 0.0149) | 0.0147 (0.00917, 0.0201) |
| t and P value | t=3.30 P=0.0010 | t=5.64 P<0.0001 | t=5.25 P<0.0001 |

^#^ : P1, P2, P3, P4 stands for1, 2, 3, ≥4 parity. Mean differences were calculated and tested at zero using paired t-test. For pair matching, age difference was set at less than or equal to 1.
